# Behavioral Aversion to AITC Requires Both *Painless* and *dTRPA1* in *Drosophila*

**DOI:** 10.3389/fncir.2018.00045

**Published:** 2018-07-03

**Authors:** Samantha J. Mandel, Madison L. Shoaf, Jason T. Braco, Wayne L. Silver, Erik C. Johnson

**Affiliations:** ^1^Department of Biology, Wake Forest University, Winston-Salem, NC, United States; ^2^Center for Molecular Communication and Signaling, Wake Forest University, Winston-Salem, NC, United States

**Keywords:** nociception, *Drosophila*, TRPA, allyl isothiocyanate, aversion

## Abstract

There has been disagreement over the functional roles of the *painless* gene product in the detection and subsequent behavioral aversion to the active ingredient in wasabi, allyl isothiocyanate (AITC). Originally, *painless* was reported to eliminate the behavioral aversion to AITC, although subsequent reports suggested that another trpA homolog, *dTRPA1*, was responsible for AITC aversion. We re-evaluated the role of the *painless* gene in the detection of AITC, employing several different behavioral assays. Using the proboscis extension reflex (PER) assay, we observed that AITC did not reduce PER frequencies in *painless* or *dTRPA1* mutants but did in wild-type genotypes. Quantification of food intake showed a significant decline in food consumption in the presence of AITC in wild-type, but not *painless* mutants. We adapted an oviposition choice assay and found wild-type oviposit on substrates lacking AITC, in contrast to *painless* and *dTRPA1* mutants. Lastly, tracking individual flies relative to a point source of AITC, showed a consistent clustering of wild-type animals away from the point source, which was absent in *painless* mutants. We evaluated expression patterns of both *dTRPA1* and *painless*, which showed expression in distinct central and peripheral populations. We identified the transmitter phenotypes of subsets of *painless* and *dTRPA1* neurons and found similar neuropeptides as those expressed by mammalian trpA expressing neurons. Using a calcium reporter, we observed AITC-evoked responses in both *painless* and *dTRPA1* expressing neurons. Collectively, these results reaffirm the necessity of *painless* in nociceptive behaviors and suggest experiments to further resolve the molecular basis of aversion.

## Introduction

Organisms must be able to perceive the quality of their environment and distinguish beneficial food sources (appetitive cues) from ones that are potentially damaging (aversive cues). Many of the molecular mechanisms that underlie either of these sensory capacities have been determined and the receptors for many different nociceptive compounds have been identified. Prominent amongst these receptor molecules are members of the transient receptor potential (Trp) channel family, based on their activation by a wide pharmacological spectrum (Latorre et al., 2009). While Trp channels are the known molecular receptors for many different compounds (Ramsey et al., 2006; Doerner et al., 2007; Kwon et al., 2010; Soldano et al., 2016), the overall architecture of nociception at a neural circuit level is not completely understood.

In the genetically tractable model system, *Drosophila*, there have been conflicting reports concerning the molecular nature of behavioral aversion to the principal nociceptive agent present in horseradish, allyl isothiocyanate (AITC). Specifically, an initial investigation of the *painless* gene, which shares homology with the mammalian TRPA class of channels, reported that this gene product was required for behavioral aversion to noxious temperatures (Tracey et al., 2003). Based on the apparent molecular homology of *painless* and mammalian TRPA channels, a subsequent report identified that *painless* mutations caused a lack of behavioral aversion to AITC, which is an established agonist of mammalian TRPA channels (Al-Anzi et al., 2006). However, functional expression of the *painless* gene in HEK-293 cells failed to confer AITC sensitivity, but did confer heat sensitivity (Sokabe et al., 2008). Subsequently, the behavioral role of *painless* in AITC aversion was questioned, as a study performed by Kang et al. (2010) suggested that *painless* was not required for behavioral aversion to AITC, but rather a different gene encoding a distinct TRPA homolog, *dTRPA1*, (Rosenzweig et al., 2005; Hamada et al., 2008) was an essential component for behavioral aversion to AITC. We suspected that these reported differences may have stemmed from technical issues in the experimental details between these groups, or alternatively, by an unappreciated complexity in the detection and subsequent aversion to AITC.

We performed experiments to reevaluate the roles of *painless* and *dTRPA1* in the physical detection of AITC and the potential contribution of this gene to chemical nociception. We performed a systematic evaluation of the proboscis extension reflex (PER) assay, which is the standard of the field in assessing *Drosophila* aversive behaviors, and adapted a feeding assay to test behavioral aversion, along with developing two novel assays to test aversive behaviors. Uniformly, these different behavioral assays show that both *painless* and *dTRPA1* gene products are required for aversive behaviors to AITC. We also evaluated expression patterns of both TrpA homologs and identified transmitter phenotypes of *painless* and *dTRPA1* cells in the adult ventral nerve cord. Using these identifiable cells, we observed AITC evoked responses in both *painless* and *dTRPA1* neurons. Collectively, our results suggest a complex neural circuit which requires both of these gene products (*painless* and *dTRPA1*), for aversion to AITC.

## Materials and Methods

### *Drosophila* Stocks and Husbandry

All flies were maintained in an incubator at 25°C and under a 12:12 LD (light-dark) cycle. Flies were cultured on a standard molasses-malt-cornmeal-agar-yeast medium and housed in uncrowded conditions. All transgenes were backcrossed to the *w*^1118^ background for five generations. The specific *Drosophila* lines used in this study (followed by Bloomington Stock # (BL#) if applicable) were the *pain*^01^ (Tracey et al., 2003; BL#-27895), *dTRPA1*^01^ (Kwon et al., 2010), UAS-ork (Nitabach et al., 2002; BL#-6586), pain-GAL4 (Tracey et al., 2003; BL#-27894), dTRPA1-GAL4 (Hamada et al., 2008), UAS-GCaMP5 (BL#-, UAS-TrpV1; Xu et al., 2010), and UAS-mCD8-GFP (BL#-5130) and *w*^1118^. The *painless* mutant was generously donated by Dr. Dan Tracey, the dTRPA1-GAL4 and mutant lines were generously donated by Dr. Paul Garrity, and the UAS-TrpV1 line was generously donated by Dr. Ping Shen, all other stocks were received from the Bloomington Stock Center.

### Proboscis Extension Response Assay (PER)

Three to 5-day old flies were separated by sex and starved for a 24-h period on 2% agar medium at 25°C. Flies were anesthetized with CO_2_, glued on their dorsal side to a petri dish, and allowed to recover for 2 h in humidified conditions at 25°C before being tested. For each genotype, three replicates of 60 males and 60 females were tested. Flies that did not show signs of movement after the 2-h recovery period were omitted from the trial. A cotton swab was used to deliver test compounds to the flies. The swab was dipped into the solutions and then contacted the flies’ legs for 3 s. Water was used to create a baseline for measuring the proboscis reflex for each genotype. All of the combinations of sucrose (1%, 2%, 5%, 10%) and AITC (1 mM, 2 mM, 5 mM, 10 mM) test solutions were used to measure the effect of varying concentrations of sucrose and AITC. Sucrose solutions were corrected to contain equimolar amounts of DMSO solvent to serve as vehicle controls. Upon presentation of the solution, an extension of the proboscis was given a score of 1, while lack thereof was given a score of 0. The percent of flies that extended their proboscis was calculated for each genotype. Statistical analyses were conducted in GraphPad using a one-way analysis of variance (ANOVA) test within genotypes and a Tukey’s test was employed to compare results to the control solution.

### Two-Choice Capillary Feeding Assay (CAFE)

As a second independent measure of aversion, we adapted the CAFE assay (Ja et al., 2007). This assay quantitatively measures aversion by calculating the exact amount of food intake of the flies. Three to 5-day old flies were separated by sex, and then starved for a 24-h period on 2% agar medium at 25°C. The flies were anesthetized using CO_2_ and five male flies or five female flies were placed in each CAFE apparatus and kept in an incubator at 25°C for 3 h beginning at ZT0 (Zeitgeber time 0 (lights on)) and tested at ZT3. For each genotype, 10 replicates of males and females were tested. The CAFE apparatus consisted of an inner vial with holes in the bottom. Two calibrated 5 μL capillary tubes (Drummond Scientific Company) were inserted into the inner vial: one containing the control solution and the other the test solution. The control solution was 1% sucrose. The test solution capillary contained sucrose (1%, 2%, 5%, 10%) and AITC (1 mM, 2 mM, 5 mM, 10 mM). Sucrose solutions were corrected to contain equimolar amounts of DMSO solvent to serve as vehicle controls. Mineral oil (Sigma) was added to the top of each capillary to prevent evaporation of the solutions. After 3 h, the amount consumed in each capillary tube was measured and the percent of test solution consumed was calculated. Statistical analyses were conducted with GraphPad using a *t*-Test between sucrose and AITC containing solutions for each genotype.

### Oviposition Assay

Three-day old female and male flies were placed together on a split plate petri dish (Genesee Scientific), with different media placed on each side as modified from Yang et al. (2015). The plates were then placed in an incubator at 25°C for 24 h. For each genotype, 10 replicates consisting of 10 females and four males were tested. Males were used in the assay to increase the amount of eggs laid in the 24-h time period. On the split-plate, one half contained the control solution (minimal media (Merico et al., 2007) containing apple juice) and the other half contained the experimental solution (minimal media + 1 mM AITC). Sucrose solutions were corrected to contain equimolar amounts of DMSO solvent to serve as vehicle controls. After 24 h the total number of eggs laid on each side was counted. Statistical analyses were conducted using GraphPad using a wilcoxon signed rank test.

### Point Source Assay

Three to 5-day old flies were separated by sex before being placed in the locomotion apparatus. For each trial, one fly was placed in a glass tube (10 cm length) with two cotton balls at each end, one saturated with vehicle solution and the other saturated with 2 mM AITC. For each genotype, 10 replicates of males and 10 replicates of females were tested. EthoVision software was used to track the movement of each fly for 10 min, and the total time spent in the two zones, (vehicle and 2 mM AITC) and the average distance from AITC point source was calculated. The zones were defined as half the distance of the tube. Statistical analyses were conducted using GraphPad using One-Way ANOVA Tests followed by a Tukey’s *post hoc* analysis.

### Immunocytochemistry

All tissues were dissected in phosphate buffered saline (PBS), rapidly fixed in a 4% paraformaldehyde 7% picric acid (PF/PA) solution for 1 h at room temperature as previously described (Zhao et al., 2010). Briefly, tissues were washed in PBS, and mounted for confocal imaging. Preparation of the legs consisted of 1 h of fix in a PF/PA solution at room temperature, six 10-min washes in PBS-0.3% Triton X (PBS-Tx), 1 h in a 1:4 dilution of a 30% hydrogen peroxide stock solution to distilled water, followed by six 10-min washes in PBS-Tx. The legs were then dehydrated in 70% glycerol and mounted onto a slide with FluoroGel mounting medium. Fluorescently labeled legs were imaged at 40× using a Zeiss LSM 710 confocal microscope. Optical sections were collected at 0.95 NA objective at 0.351-μm intervals. Next, Z-stacks were taken and compiled into maximum intensity projections. Spectral separation was used with the images of the legs to remove autofluorescence of the cuticle. For co-staining experiments, tissues were blocked overnight and incubated with primary antibody overnight at 4°C. Anti-DH31 and anti-leucokinin were generous gifts from Dr. Jan Veenstra, and both were used at 1:1000 dilutions (Johnson et al., 2005). Brains were washed and a Cy-3 conjugated anti-rabbit secondary antibody (Jackson Laboratories) was applied overnight at 1:1000 dilution. Tissues were then mounted and viewed on a Zeiss 710 confocal microscope. The images were exported as TIF files and stitched together in PowerPoint to form a picture of the entire structure (brain, leg, or labellum).

### Live Cell Imaging

Adult progeny from flies carrying either the *painless-GAL4* or the *dTRPA1-GAL4* transgene crossed to the UAS-GCaMP lines were dissected and placed in AHL (adult hemolymph-like; Soldano et al., 2016) solution. Following a 15-min incubation period, explanted brains were then viewed on a Zeiss LSM 710 confocal microscope (see Braco et al., 2012 for additional details). Images were collected every 15 s for experiments to minimize photobleaching of GCaMP fluorescence, from a collapsed Z stack. Following experiments, 3 M KCl was applied to evoke cell depolarization as a measure of cell viability; only cells which showed KCl evoked cell increases in GCaMP fluorescence were used for analysis. Confocal settings were identical for all experiments. A region of interest was manually drawn for each cell and total values for pixel intensity were assessed. Values were exported in Excel and normalized to baseline levels. Ten replicates for each treatment and genotype were analyzed.

## Results

### Painless and dTRPA1 Genes Are Required for Behavioral Aversion to AITC

To re-evaluate the potential roles of the *painless* gene in the behavioral aversion to AITC, we initially tested previously reported mutants in the proboscis extension response assay (PER). This assay is largely considered a standard method to evaluate both aversive and appetitive behaviors (e.g., Huetteroth et al., 2015), however, there is a great deal of experimental variation in conducting this assay from group to group. Consequently, we modified the assay with the goals of a comprehensive evaluation of the technique to help standardize the methodology. One experimental difference in PER is the elimination of results from animals that showed a positive response to water, following the experimental test (Al-Anzi et al., 2006). The rationale for doing so is based on the argument that a positive response to water reflected a state of thirst, as opposed to hunger. We chose not to do this, as we maintain that a positive PER response is based on an appetitive stimulus, and aversion should curtail or influence the likelihood of PER independent of the nature of the appetitive stimulus (water or food). We did place animals in a humidified environment prior to testing and observed no differences in the PER frequencies to water alone (Supplementary Figure S1). Another experimental difference between groups is one group presented multiple offerings of the aversive stimulus, and the methods of pooling behavioral responses. Additionally, different concentrations of appetitive and/or aversive stimuli have been employed and other experimental variations reflect differences in scoring the response.

**Figure 1:**
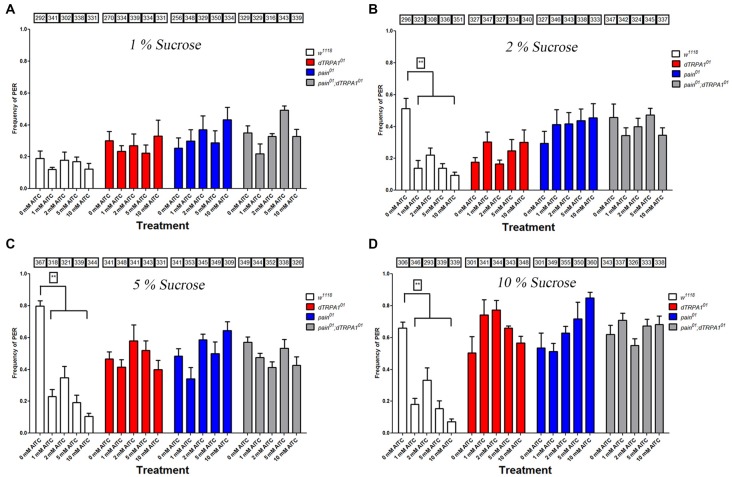
Both *painless* and *dTRPA1* mutants display no reduction in proboscis extension to allyl isothiocyanate (AITC). Frequencies of proboscis extension reflex (PER) are plotted for the *w*^1118^, *pain*^01^, *dTRPA1*^01^ and *pain*^01^; *dTRPA1*^01^ double mutant lines for 0 mM, 1, 2, 5 and 10 mM AITC tested with different sucrose concentrations (1% plotted in panel **A**, 2% in Panel **B**, 5% in Panel **C** and 10% in Panel **D**). When tested at 1% sucrose, no differences in PER frequencies were observed at any AITC concentration for any genotype. At higher sucrose concentrations, higher PER frequencies were observed for the control solution (0 mM AITC) for each genotype. Addition of AITC lead to a significant decrease in PER frequency for the control genotype (*w*^1118^), whereas in the other genotypes, no such decrease in PER frequency was observed. Numbers in the boxes refer to the total number of individual flies tested under the corresponding experimental condition. **Indicates significance at *p* < 0.001 (Tukey’s *post hoc* test, one-way analyses of variance (ANOVA)).

We chose to test PER frequencies under different sucrose and AITC levels, as the previous discrepancies may have resulted from different concentrations tested in previous reports. We found that in wild-type animals, PER levels increased as a function of sucrose concentration. At relatively low sucrose levels (1%), we found nominal levels of PER responses, which were not significantly different than PER responses to water as a test stimulus. At this level of sucrose, we were unable to detect aversion to AITC in wild-type animals due to the low frequency of the appetitive response to sucrose. However, at all other sucrose concentrations tested (2%–10%), PER frequencies were sufficiently high to detect decreased responses to solutions containing AITC (Figure 1). This decreased PER response was observed at all levels of AITC (1–10 mM). In contrast, PER frequencies for the *pain*, *dTRPA1* and *pain;dTRPA1* double mutant lines were unchanged by the presence of AITC (Figure 1). We also confirmed previous reports that both *painless* and *dTRPA1* mutants have a specific defect in aversion to AITC and not a general defect, as both genotypes decreased PER frequencies to salt and quinine (Al-Anzi et al., 2006; Kang et al., 2010).

**Figure 2:**
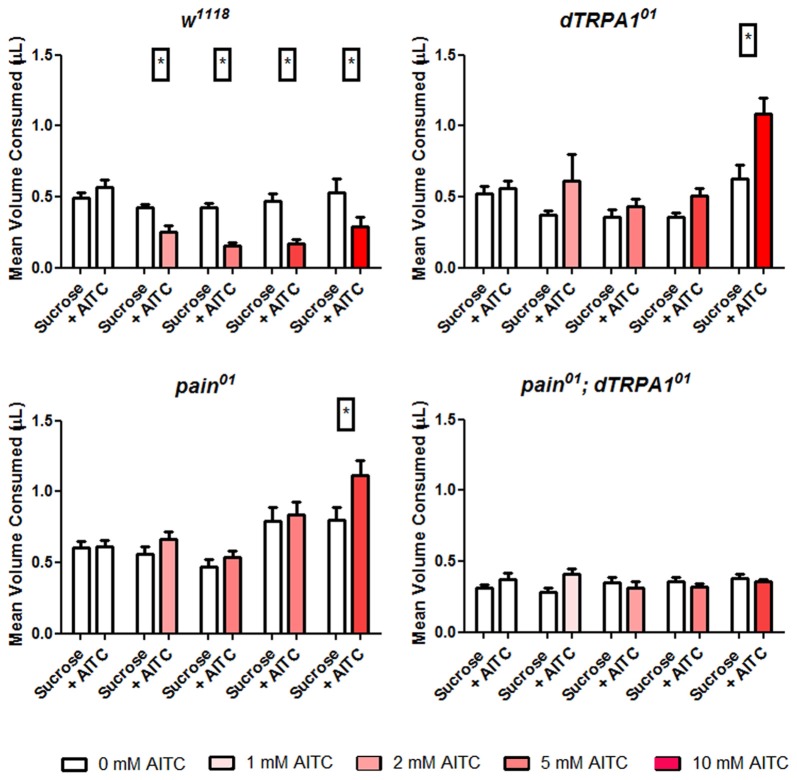
Both *painless* and *dTRPA1* mutants show no aversion to food consumption containing AITC. Total food intake was measured in a modified CAFE assay that incorporated two capillary feeders, one containing sucrose and the other containing sucrose and AITC and tested for 10 replicates of five individuals. Under control conditions (i.e., 0 mM AITC), equal volumes of solution were consumed for all genotypes. However, in the control (*w*^1118^) genotype, there was a significant reduction (*P < 0.05 *T*-test) in the volume of food consumed from the feeder containing AITC, and this reduction was observed under all AITC concentrations. In the *pain*^01^, *dTRPA1*^01^ and double mutants, there was no reduction of food consumed from the AITC feeder.

Considering that the PER assay relies on the subjective scoring of a positive response, we adapted the CAFE assay to further test the roles of the *painless* gene in the behavioral aversion to AITC. The CAFE assay quantitatively measures the volume of food intake (Ja et al., 2007) and we modified this assay for a two-choice assay. To maximize the volume that animals were consuming, we used a 1% sucrose solution and tested this against feeders containing 1% sucrose and AITC (1, 2, 5 and 10 mM). Wild-type animals showed a significant reduction in the volume consumed from feeders containing AITC (*P* = 0.001 One Way ANOVA). In contrast, both the *painless* and *dTRPA1* mutant lines showed no such reduction in the volumes consumed from AITC containing feeders (Figure 2). We note that at high concentrations of AITC, both the *painless* and *dTRPA1* mutant consume more food containing AITC, which could reflect non-specific activation of gustatory receptors at these high concentrations of AITC.

**Figure 3:**
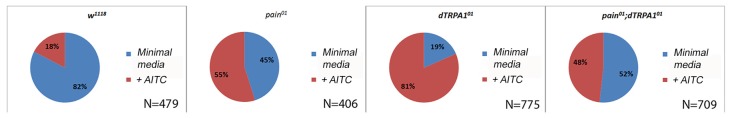
The *painless* mutant shows no avoidance to AITC for oviposition choice. Oviposition sites were measured on a split plate containing equal areas of media and media plus 1 mM AITC. For the control genotype (*w*^1118^), there were significantly more eggs laid on the non-AITC side (*P* = 0.005 wilcoxon signed rank test), whereas for the *pain*^01^ and double mutant lines there was no significant preference for one side. In the *dTRPA1*^01^ line, there is a significant preference for the solution containing AITC (*P* = 0.002 wilcoxon signed rank test). N’s refer to the total number of eggs counted.

Both the PER and CAFE assays test aversion within the context of feeding, and thus require animals to be starved prior to testing. While we observed no differences in PER and CAFE response in our different mutant genotypes, there may have been differences in starvation sensitivity (Johnson et al., 2010) which could have explained the conflicting results in the literature. Therefore, we sought to test aversion in multiple behavioral and physiological contexts. Oviposition represents an important decision by females, as the nutritive value of the food directly impacts her offspring’s survival rate (Schwartz et al., 2012). We tested oviposition preference utilizing a split Petri dish which possessed a minimal media on one side, and minimal media with 1 mM AITC on the other side. Females were allowed to lay eggs overnight and we observed a strong preference for oviposition on the AITC free side in wild-type animals (Figure 3). The *painless* mutant females showed no preference for oviposition site whereas the *dTRPA1* mutant females showed a strong preference for laying eggs on the AITC substrate (*P* = 0.005, wilcoxon signed rank test). The double mutant line showed no preference for oviposition site.

**Figure 4:**
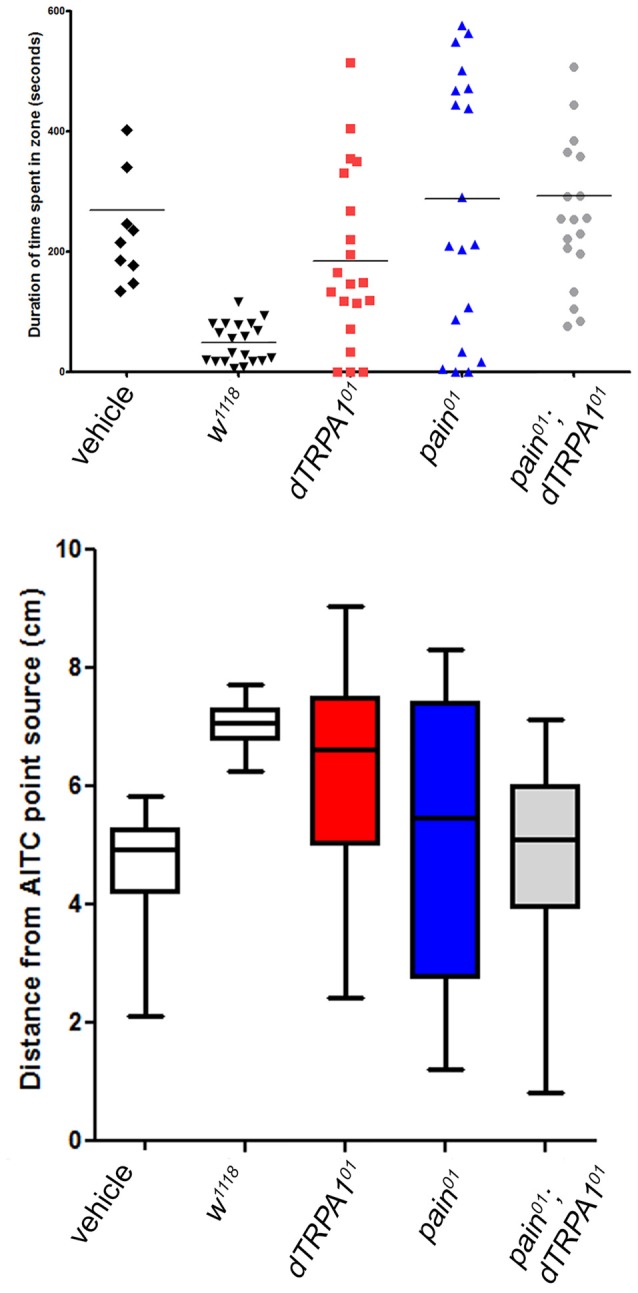
Both *painless* and *dTRPA1* mutants show no avoidance to an AITC point source.** (A)** Measurements of the average total duration in the AITC zone (defined as 5 cm (or halfway) from the AITC point source) are plotted for each genotype. In the control genotype (*w*^1118^) with no AITC, the duration spent in one zone vs. the other is equal, whereas in the same genotype tested with AITC, the animals spent significantly less time in the AITC zone (*P* = 0.001 One Way ANOVA; each symbol represents a single animal trial and the lines represents the mean for all animals). For all the other genotypes, there was no significant difference in the time spent in one zone as compared to the other. **(B)** The average distance from the AITC point source (center line) and standard deviation are plotted for the same genotypes. The *w*^1118^ animals tested with AITC were significantly different than 5 cm away (reflecting a random distribution; *P* = 0.001, One-Way *T*-Test), whereas the other gentoypes showed no significant difference from the center point.

We also reasoned that aversive behavior should be evident as avoidance of a point source of a potential irritant. Consequently, we placed animals in a 10 cm long glass tube that had a cotton plug saturated with vehicle on one end and AITC-saturated plug on the other. Flies were recorded for a 10-min period and the mean duration of time spent in the vehicle zone or the AITC zone, and the average distance from the AITC point source were evaluated. For wild-type animals, there was a clear bias away from the AITC point source (Figure 4). In contrast, both *painless* and *dTRPA1* mutant animals spent more time in the AITC zone and the average distance was more varied (*P* = 0.001, one-way ANOVA).

### Expression of dTRPA1 and Painless in Multiple Tissues Appear Distinct

**Figure 5:**
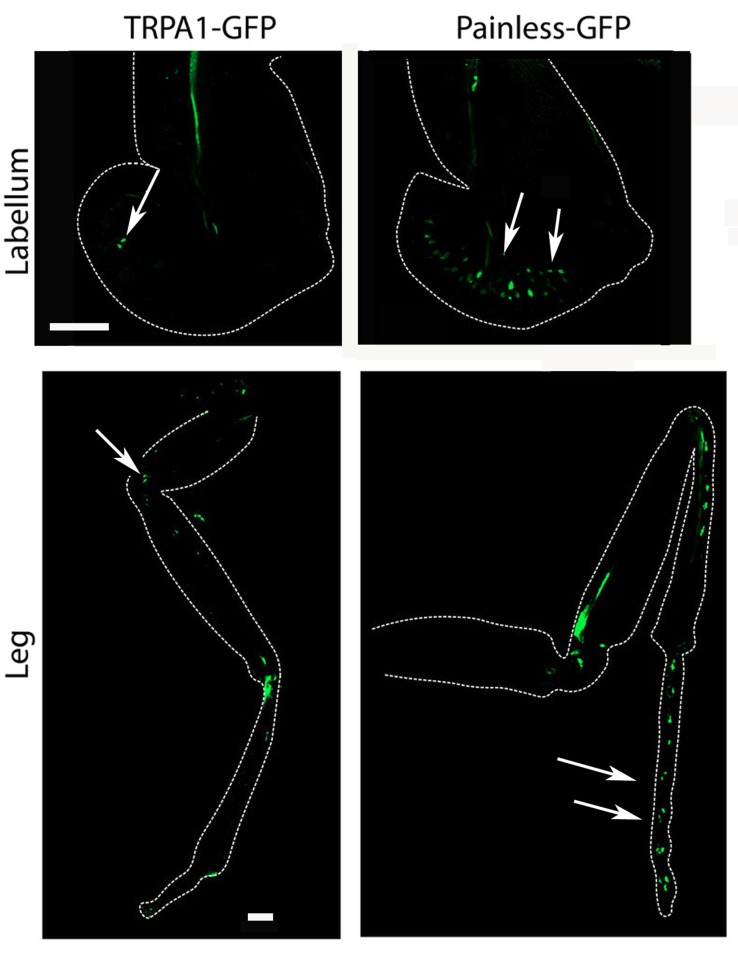
Expression of the *painless*-GAL4 and *dTRPA1*-GAL4 lines appears to be expressed in different tissues. We introduced a membrane-tethered GFP variant under the control of the *painless*-GAL4 and *dTRPA1*-GAL4 drivers and examined different peripheral tissues and the central nervous system. Expression of *painless*-GAL4 in the labellum (right) is widely expressed in multiple sensory neurons (white arrows) compared to the *dTRPA1*-GAL4 (left) in the same structure, in which only a cluster of a few neurons were detectable (white arrow). Expression of *painless*-GAL4 (left) in the leg also appears to be widespread in multiple neurons in the distal portions of the leg (white arrows) compared to the *dTRPA1*-GAL4 (right) expression pattern that is more proximal and in fewer cells (white arrows).

**Figure 6:**
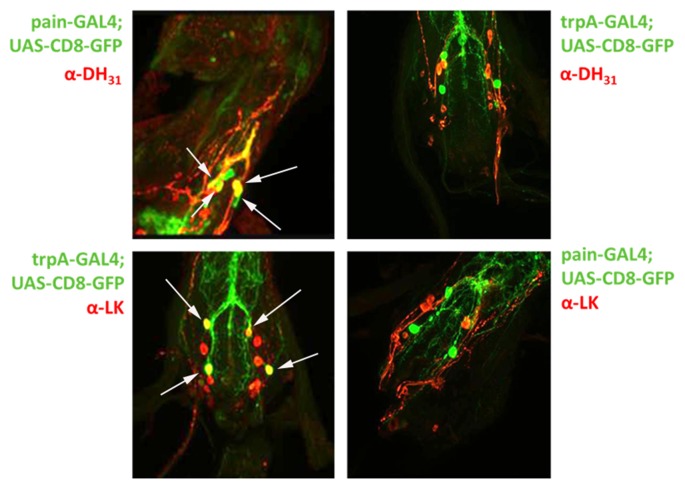
Co-expression of *painless* and *dTRPA1* with the DH31 and Leucokinin neuropeptides in the adult CNS. Given the expression patterns presented in Supplementary Figure S2, we tested the possibility that the neurons in the adult ventral nerve cord were expressing both *painless* and *dTRPA1*. To test this hypothesis, we used antibodies against mutiple peptides and found that four *painless* GAL4 positive neurons are specifically labeled (white arrows) with an antibody against the DH31 peptide (top left), whereas four *dTRPA1* GAL4 positive neurons (white arrows) are specifically labeled by antisera against the Leucokinin peptide (bottom left). Conversely, DH31 postive immunosignals do not label *dTRPA1* neurons (top right) and the *painless* neurons are not labeled with the leucokinin antibody (bottom right). There are additional neuropeptide staining cells present in the ventral nerve cord, but there are only four neurons that are labeled by the *painless* and *dTRPA1* drivers. The GFP that is present in the merge reflects processes of these neurons vs. the soma.

Our results clearly demonstrate that both the *painless* and *dTRPA1* gene products are required for normal aversive behaviors to AITC and suggest multiple questions to understand the underlying mechanism. The obvious question of whether *painless* and *dTRPA1* are expressed in the same cells or in different cell populations would implicate potentially different mechanisms. To evaluate expression patterns, we employed previously reported GAL4 drivers that recapitulate the expression patterns of *dTRPA1* and *painless*. Notably, these driver lines were used to genetically rescue the defects associated with each mutant (Tracey et al., 2003; Kang et al., 2010). It had been previously reported that the *dTRPA1*-GAL4 was not expressed in the adult leg, and prominently expressed in the labral sense organ, and that the *pain*-GAL4 directed expression in the leg, labellum, and throughout the nervous system (Tracey et al., 2003). We used spectral separation, as these peripheral tissues possess substantial autofluorescence, and we observed similar expression patterns as those previously reported. We note *dTRPA1*-GAL4 expression in the leg, but only in proximal segments, compared to the distal expression of *painless* (Figure 5). In the labellum, we observed similar patterns with *dTRPA1*-GAL4 driving expression in proximal areas, and *painless*-GAL4 driving expression comparatively in more distal sections of the labellum. Within the adult ventral ganglion, we observed *painless*-expressing neurons in the same vicinity as the *dTRPA1*-GAL4-expressing cells (Supplementary Figure S2). Given that there are an equal number of cells (4) within the second thoracic segment of the ventral nerve cord that are labeled in these drivers, we attempted to identify markers for the different *painless* and *dTRPA1* adult cell populations and ultimately to test to see if these cells were expressing both genes or represented different cell populations. We used several different antisera for neuropeptides and found that *painless*-expressing cells in the adult ventral nerve cord were immunolabeled with the DH31 antisera. These four cells were in close proximity to *dTRPA1* driven expression, however, DH31 antisera failed to label the *dTRPA1* population (Figure 6). We did find that these *dTRPA1* cells were immunolabeled by antisera targeting the leucokinin neuropeptide. The significance of these findings is that DH31 shares homology with mammalian CGRP (Johnson et al., 2005), and that leucokinin shares homology with mammalian Substance P (Johnson, 2006), and that in the mammalian trigeminal system, CGRP and Substance P are the principal transmitters and are coexpressed with mammalian TRPA1. While we cannot exclude that *painless* and *dTRPA1* are coexpressed, it appears that peripheral expression of these two driver lines are distinct, and also appear to be different in the CNS.

### Painless and dTRPA1 Neurons Are Both Necessary but Not Sufficient for Aversion

**Figure 7:**
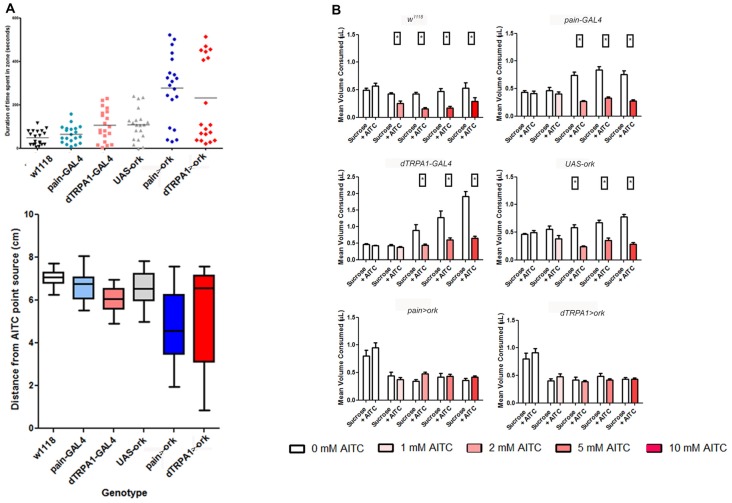
Electrical suppression of either *painless* or *dTRPA1* neurons leads to a loss of aversion to AITC. Considering that these drivers have been previously shown to rescue the phenotypes associated with loss of function alleles of both *painless* and *dTRPA1*, we tested whether these different drivers capture relevant cellular populations. We introduced the potassium leak channel, *Δork*, to *painless* and *dTRPA1*-expressing cells and measured aversive behaviors in the different assays. **(A)** Electrical silencing of either population showed no discrimination in the point source assay, in contrast to control genotypes which spend significantly more time in the non-AITC zone, and maintained a greater average distance away from the AITC point source (One way ANOVA, *P* < 0.001; *N* = 20 animals). **(B)** Similar results were obtained in food intake quantities, in which control genotypes consumed signficantly less AITC containing food (*P* < 0.05 *T*-test). In contrast, silencing of *painless*-expressing or *dTRPA1*-expressing cells consumed equal quantities of AITC and control food solutions (*N* = 50 total animals).

Given that the expression patterns of these different driver lines appear distinct, we next asked whether both cell populations were necessary for behavioral aversion to AITC. To test this hypothesis, we examined the effects of electrical inactivation of neurons expressing these different GAL4 elements on behavioral aversion to AITC. We introduced the potassium leak channel, ORK (Nitabach et al., 2002) to *painless* GAL4 or *dTRPA1* GAL4-expressing cells and tested them in our different behavioral assays. We observed that in all four assays, that electrical suppression of these cells using these different drivers all caused a loss of aversion to AITC. Specifically, equal volumes of a sucrose solution and a sucrose + AITC solution were consumed in animals with *painless* or *dTRPA1* silenced neurons, whereas in all of the parental controls, there was a clear preference for the sucrose only solution (Figure 7). Likewise, in the point source assay, control animals showed a significant duration and average distance away from the point source (*P* < 0.001, one-way ANOVA), which was lacking in animals with electrically inactivated neurons. The greater variance seen in electrical suppression of these neuronal groups are consistent with the expectation, that aversion to AITC was lost. Furthermore, the expected decline in PER frequencies in the presence of AITC in animals with *painless* or *dTRPA1* suppressed neurons was absent and aversion for female oviposition was likewise lost in animals with suppressed *painless* or *dTRPA1*-expressing neurons (Supplementary Figure S3).

**Figure 8:**
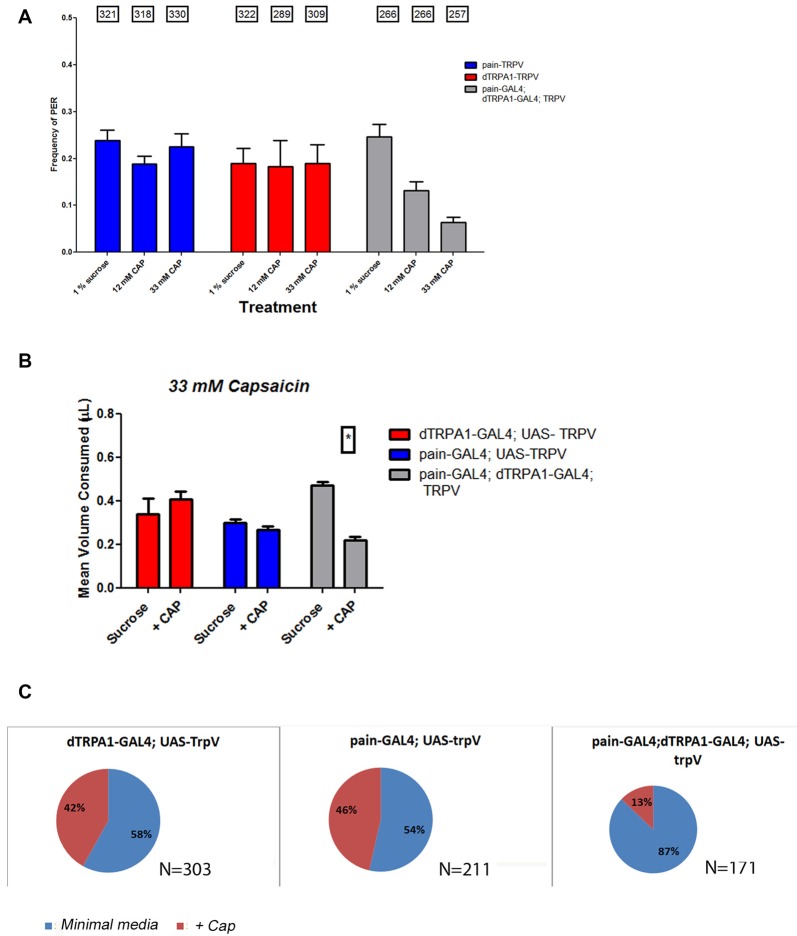
Introduction of the mammalian TRPV1 channel to both *painless* and *dTRPA1* populations confers aversion to capsaicin. To further examine the circuitry associated with aversion, we introduced the mammalian TrpV1 channel to either *painless* or *dTRPA1*-expressing cells and measured their response to capsaicin, a TrpV1 agonist. Expression of TrpV1 in either *painless* or *dTRPA1* cells produced no change in PER frequencies in the presence of capsaicin **(A)**, but TrpV1 introduction to both cell populations, did reduce the frequency of PER response to capsaicin. Similar results were obtained in food consumption **(B)**, in which TrpV1 introduction to either cell population alone produced no aversion to capsaicin, but did once TrpV1 was added to both populations. Lastly, oviposition preference was unaltered in *painless* >TrpV1 or *dTRPA1* >TrpV1 genotypes, but was significantly changed (*P* < 0.05 wilcoxon signed rank test) in TrpV1 introduction to both cell populations **(C)**.

Our behavioral results implicate that both *painless* and *dTRPA1* are necessary for behavioral aversion. We next wanted to test the sufficiency of either of these channels for aversive behaviors. To test this, we introduced the TrpV1 channel (Caterina et al., 1997; Xu et al., 2010) which is specifically activated by capsaicin and is not present in *Drosophila*, to either *painless* expressing or *dTRPA1*-expressing cells or simultaneously to both groups. In both the PER and two-choice CAFE assay, introduction of TrpV1 to these cell populations failed to confer aversion to capsaicin (Figure 8), suggesting that activation of these cellular populations is insufficient for aversive behaviors. In contrast, simultaneous introduction of TrpV1 to both cell populations led to aversive behaviors. Likewise, expression of TrpV1 in either subgroup alone did not confer aversion to capsaicin, however, capsaicin was avoided by females expressing TrpV1 in both subgroups. We note the differences in capsaicin in the control genotypes, with some controls appearing to show preference vs. no preference to the compound, however, it is clear that there is no aversive behavior elicited in any of these assays by the introduction of TrpV1 to either subgroup, and may reflect activation of gustatory receptors by capsaicin.

### dTRPA1 and Painless-Expressing Cells Exhibit AITC Evoked Calcium Changes

**Figure 9:**
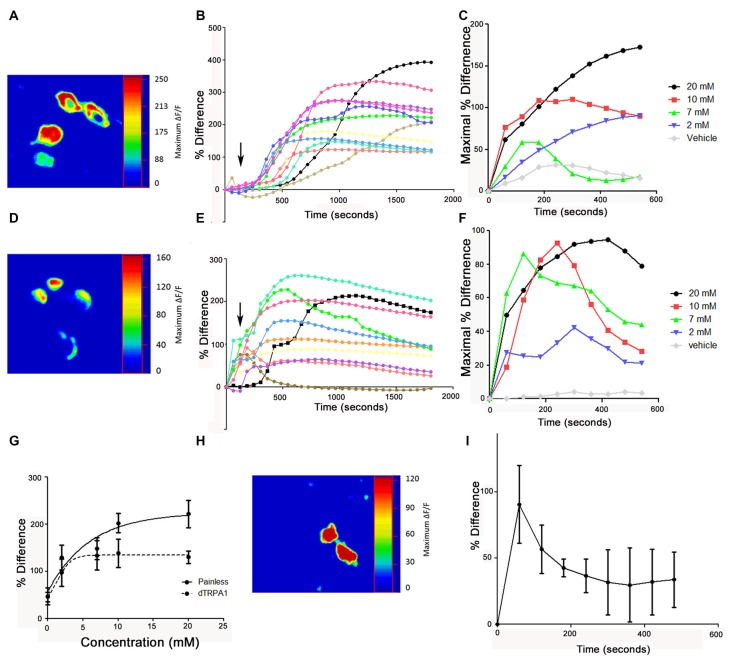
Application of AITC elicits calcium increases in both *painless* and *dTRPA1*-expressing neurons.** (A)** Representative heat maps of *painless*-expressing neurons in the adult VNC following 2 mM AITC application. Heat maps show the percent change between basal and maximal responses and are from the four DH31-expressing neurons in the adult VNC. **(B)** Representative cellular responses and timecourses from 11 different *painless*-expressing neurons in response to 2 mM AITC from five different brains. **(C)** Timecourse of responses of *painless* neurons to different concentrations of AITC (arrows indicate addition). Standard deviations are not shown for figure clarity. **(D)** Representative heat maps of *dTRPA1*-expressing neurons in the adult VNC following 2 mM AITC application. Heat maps show the percent change between basal and maximal responses and are from the four LK-expressing neurons in the adult VNC. **(E)** Representative cellular responses and timecourses from 10 different *dTRPA1*-expressing neurons in response to 2 mM AITC from four different brains **(F)**. Timecourse of responses of *dTRPA1* neurons to different concentrations of AITC. Standard deviations are not shown for figure clarity. **(G)** Dose response curves plotting maximal response as a function of concentration of AITC in *painless* and *dTRPA1*-expressing neurons. **(H)** Representative heat map of DH31 expressing *painless* neurons in the adult VNC to 2 mM AITC, from *dTRPA1* mutant flies. **(I)** Mean timecourse of 2 mM AITC response of *painless* in a *dTRPA1* mutant background.

After establishing that both channels are expressed in distinct cellular populations and required for behavioral aversion to AITC, we next tested if these cells showed physiological responses to AITC. We introduced the calcium-sensitive fluorescent reporter, GCaMP to each cell population and then applied AITC. While it would be preferable to test *painless* and *dTRPA1*-expressing cells in the periphery, the autofluorescence of these structures made it impractical to do so. Consequently, we dissected adult ventral nerve cords to assess the responsiveness of the easily identifiable *painless* and *dTRPA1*-expressing neurons in the VNC. These are the neurons that coexpress the neuropeptides, DH31 and LK, respectively as they are readily identifiable by anatomical position (see Supplementary Figure S3). We observed rapid changes in GCaMP fluorescent levels in both *painless* and *dTRPA1*-expressing cells upon AITC application (Figure 9), and while there are subtle differences in the temporal architecture of the responses that we speculate reflect desensitization mechanisms, there were clear dose-dependent responses. Furthermore, these responses were reversible, as replacement of AITC solution with vehicle restored baseline fluorescent levels (data not shown). Furthermore, we note that addition of vehicle to this same population of neurons did not significantly alter GCaMP fluorescence (Supplementary Figure S4). Furthermore, the response to AITC appears specific, as neurons that do not express *painless* or *dTRPA1*, as evaluated by GAL4 expression patterns, do not respond to AITC (Supplementary Figure S4). We next tested whether the responsiveness of the *painless*-expressing cells was altered in a *dTRPA1* mutant background. Surprisingly, AITC-evoked changes in *painless* neurons were present, albeit reduced in the *dTRPA1* mutant background (Figure 9).

## Discussion

We show that the *painless* gene product is required for the behavioral aversion to AITC. We have taken a comprehensive approach that examined aversion in a number of different physiological and behavioral contexts. In all cases, independent of the specific behavioral assay or experimental condition, we find no differences between *painless* and *dTRPA1* mutants in regards to aversive behaviors. Likewise, we find that suppression of activity in either cell population completely phenocopies the *painless* or *dTRPA1* mutation, supporting the idea that both *painless* and *dTRPA1* are required for behavioral aversion to AITC. In addition to reaffirming a pivotal role for *painless* in the chemical nociception to AITC, we have developed several assays which allowed for a comprehensive evaluation of aversive behaviors. To our surprise, we found that the strength of an appetitive stimulus did not temper aversive responses. It may be that this is unique to AITC, and other compounds would show gradients of “aversion.” However, our results suggest that aversion “overrules” appetitive stimuli (Figures 1–4), which could be beneficial to the animal to avoid nociceptive agents independent of how appetitive a stimulus is.

How can we reconcile our results in the context of previous reports? We employed the proboscis-extension reflex assay (PER) as did the other groups, albeit with some notable experimental differences. We observed PER frequencies similar to those reported in Al-Anzi et al. (2006) using a similar concentration of sucrose (2%). While we did not test the exact concentration of sugar used in the later study (12%), we did not observe PER frequencies of 100% as reported in that study. We suspect that those high rates of PER responses may reflect differential starvation states, as this behavioral response is predicated upon the animals being hungry (Shiraiwa and Carlson, 2007). Likewise, the increased number of individuals we tested (a total of 26845 individuals in Figure 1) allows for stronger statistical tests. Likewise, the relative strength of the appetitive stimulus is an important factor in determining aversion, as we observed negligible PER frequencies with low amounts of sucrose, and these frequencies were equivalent to our measurements with PER using only water as a stimulus (Supplementary Figure S1), and thus we could not detect aversion to AITC under these conditions in wild-type animals. We submit that the PER assay, while easy, may not the best behavioral readout for aversion, since it depends on the loss of a response to an appetitive stimulus. This was the rationale for the development of several other behavioral assays that could directly test aversive behaviors, as well as testing aversion in different physiological contexts.

Having confirmed a role for *painless* in chemical nociception (Al-Anzi et al., 2006), the next question is how is *painless* functioning within the formation of aversive behaviors? Our evaluation of AITC evoked responses in calcium release in *painless*-expressing neurons, suggests that there is minimally additional AITC receptors independent of *dTRPA1*. Likewise, our observations that the introduction of the mammalian TrpV1 channel was required in both populations of neurons to confer aversion to capsaicin, might be explained by the presence of additional AITC sensors. However, it appears that *painless* may not be a direct target of AITC. Heterologous expression of *painless* in HEK cells failed to confer AITC sensitivity, but did confer thermal sensitivity (Sokabe et al., 2008), consistent with reports that of *painless* encodes a TRPA1 channel member modulated by temperatures (Barbagallo and Garrity, 2015). It may be that AITC binding to *painless* does occur in native tissues, or that *painless* has an indirect role in AITC evoked behavioral responses. This may occur at the molecular level, in which perhaps the *painless* channel functions as a trafficking factor for other AITC receptors, including *dTRPA1*, or perhaps as the circuit level, in which perhaps primary AITC targets in the periphery relay information to *painless*-expressing neurons centrally. While our (and others) results do not suggest coexpression of these two channels, we cannot conclusively state that there are not areas in the animal where the two channels are coexpressed. For example, the coexpression of *painless* and *dTRPA1* may occur in the larval Class IV multidendritic neurons (Zhong et al., 2012). Interestingly, the *dTRPA1* gene has been discovered to possess multiple isoforms that differ in both their expression patterns and functional properties (Zhong et al., 2012). This gene complexity makes it difficult to ascertain if the drivers we employed capture all of the relevant isoforms, although we note that both drivers had been used to genetically rescue AITC aversion (Tracey et al., 2003; Kang et al., 2010). New technologies, such as CRISPR, will be useful in firmly establishing whether these molecules are co-expressed. We have started to pursue those lines of experiments, and such information will be crucial in the evaluation of these molecules in the detection and/or circuit level organization underlying aversive behavior.

An interesting finding is that the transmitters co-expressed with the *painless* and *dTRPA1* channels are homologs of the mammalian transmitters CGRP (DH31) and Substance P (Leucokinin; Johnson, 2006). This finding suggests a conservation of neural circuits involved in nociception and suggests that in addition to the molecular homology of these TRPA channels, insight may be gained from experiments on organisms with comparatively less complex nervous systems than humans, such as *Drosophila*. Our results showing the requirement of two TRPA channel homologs is also interesting and unexpected, as *painless* and *dTRPA1* are not functionally redundant. *Drosophila* possesses two additional TPRA channel family members, *pyrexia* and *waterwitch* (Montell, 2005; Liu et al., 2007), and it would be interesting to see if either of these two channels are likewise required for chemical nociception. There is precedent for the lack of functional redundancy for a behavioral phenotype involving these same channels. The *dTRPA1*, *painless* and *pyrexia* mutants all show a lack of high temperature thermal avoidance (Tracey et al., 2003; Lee et al., 2005; Hamada et al., 2008). While these channels are activated at different temperatures, all three of them are required for thermal avoidance, implicating a lack of functional redundancy. Again, whether this stems from molecular interactions between these channels and/or alternatively or additionally, at the neural circuitry is an important question that needs to be addressed.

## Author Contributions

JB, WS and EJ: design of experiments. SM, MS and JB: performing experiments. SM, MS, JB and EJ: analyzing data. WS and EJ: drafting manuscript.

## Conflict of Interest Statement

The authors declare that the research was conducted in the absence of any commercial or financial relationships that could be construed as a potential conflict of interest.

## Supplementary Material

FIGURE S1 PER responses to water in *dTRPA1* and *painless* variants. Percentage of PER responses to water treatment alone in wild-type, *painless*, *dTRPA1*, and painless; dTRPA1 double mutants. There are no significant differences in the amount of positive PER responses to water alone (One Way ANOVA, *P* > 0.05), indicating that these genotypes aren’t differentially impacted by experimental conditions regarding hydration levels.

FIGURE S2 Expression of *painless* and *dTRPA1* in the adult CNS. Adult brains and ventral nerve cords were dissected from offspring of a *painless*-GAL4 (left) and a *dTRPA1*-GAL4 (right) cross to a UAS-mCD8-GFP fly line. Along the ventral nerve cord, note that there are four neurons that appear similar and are labeled in both *painless* and *dTRPA1*-GAL4 lines (white arrows).

FIGURE S3 Electrical silencing of *painless* and *dTRPA1* neurons impacts PER frequencies and oviposition preferences. **(A)** Electrical silencing of either the *painless*-GAL or the *dTRPA1*-GAL4 expressing neurons leads to no reduction in PER frequecies at 5% sucrose upon presentation of AITC as opposed to parental control genotypes (One way ANOVA, GraphPad). **(B)** Oviposition preference is lost in animals with silenced *painless* and *dTRPA1* neurons, as equal levels of eggs were laid on AITC substrate as control substrate.

FIGURE S4 **(A)** Addition of vehicle to *painless* expressing neurons does not significantly alter GCaMP fluorescence. Mean responses of painless expressing neurons to vehicle addition, note the photobleaching and compare to responses shown in Figure 9. **(B)** Addition of AITC to neurons that do not express *painless* or *dTRPA1* does not significantly alter GCaMP fluorescence. 2mM AITC application to neurons expressing GCaMP but not painless does not significantly change GCaMP fluorescence. Again note the photobleaching of GCaMP and compare to responses shown in Figure 9.
