# Supplementary figures and images for: Behavioral Aversion to AITC Requires Both Painless and dTRPA1 in Drosophila
Source: Front Neural Circuits. 2018 Jul 3;12:45. doi: 10.3389/fncir.2018.00045 (PMC6038230; doi:10.3389/fncir.2018.00045)

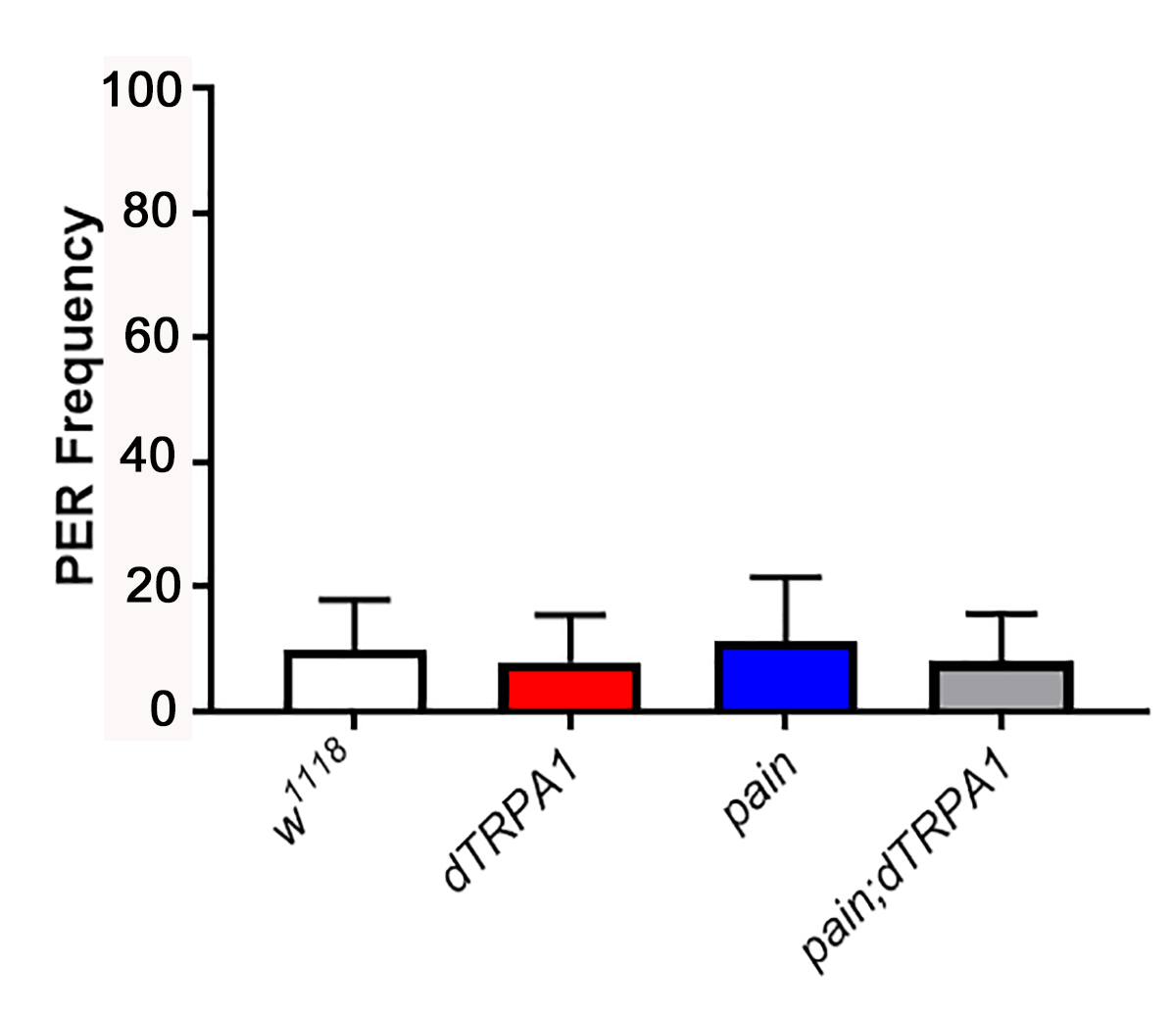

Supplement: FIGURE S1 — PER responses to water in dTRPA1 and painless variants. Percentage of PER responses to water treatment alone in wild-type, painless, dTRPA1, and painless; dTRPA1 double mutants. There are no significant differences in the amount of positive PER responses to water alone (One Way ANOVA, P > 0.05), indicating that these genotypes aren’t differentially impacted by experimental conditions regarding hydration levels. [file Image_1.TIF]

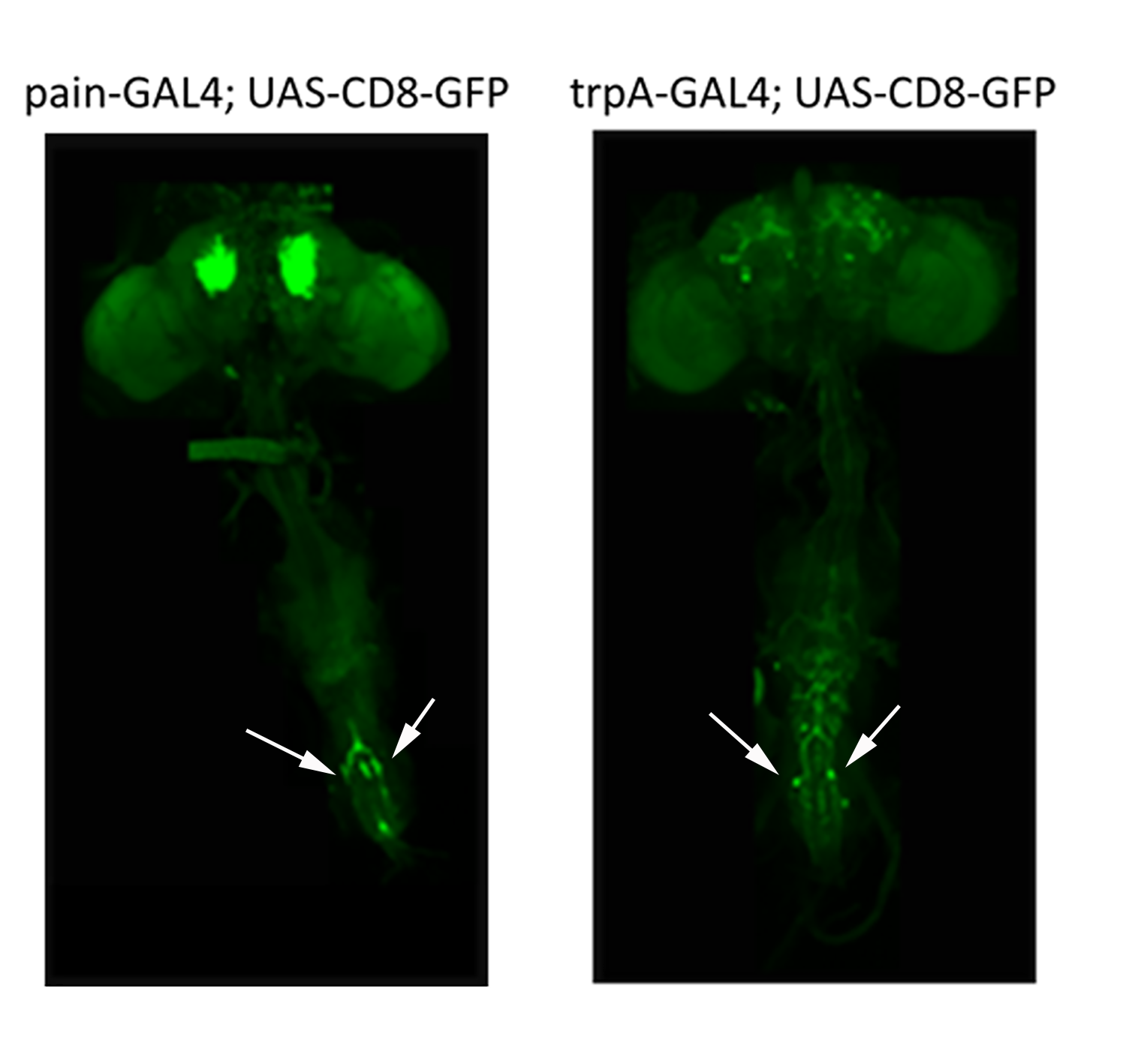

Supplement: FIGURE S2 — Expression of painless and dTRPA1 in the adult CNS. Adult brains and ventral nerve cords were dissected from offspring of a painless-GAL4 (left) and a dTRPA1-GAL4 (right) cross to a UAS-mCD8-GFP fly line. Along the ventral nerve cord, note that there are four neurons that appear similar and are labeled in both painless and dTRPA1-GAL4 lines (white arrows). [file Image_2.TIF]

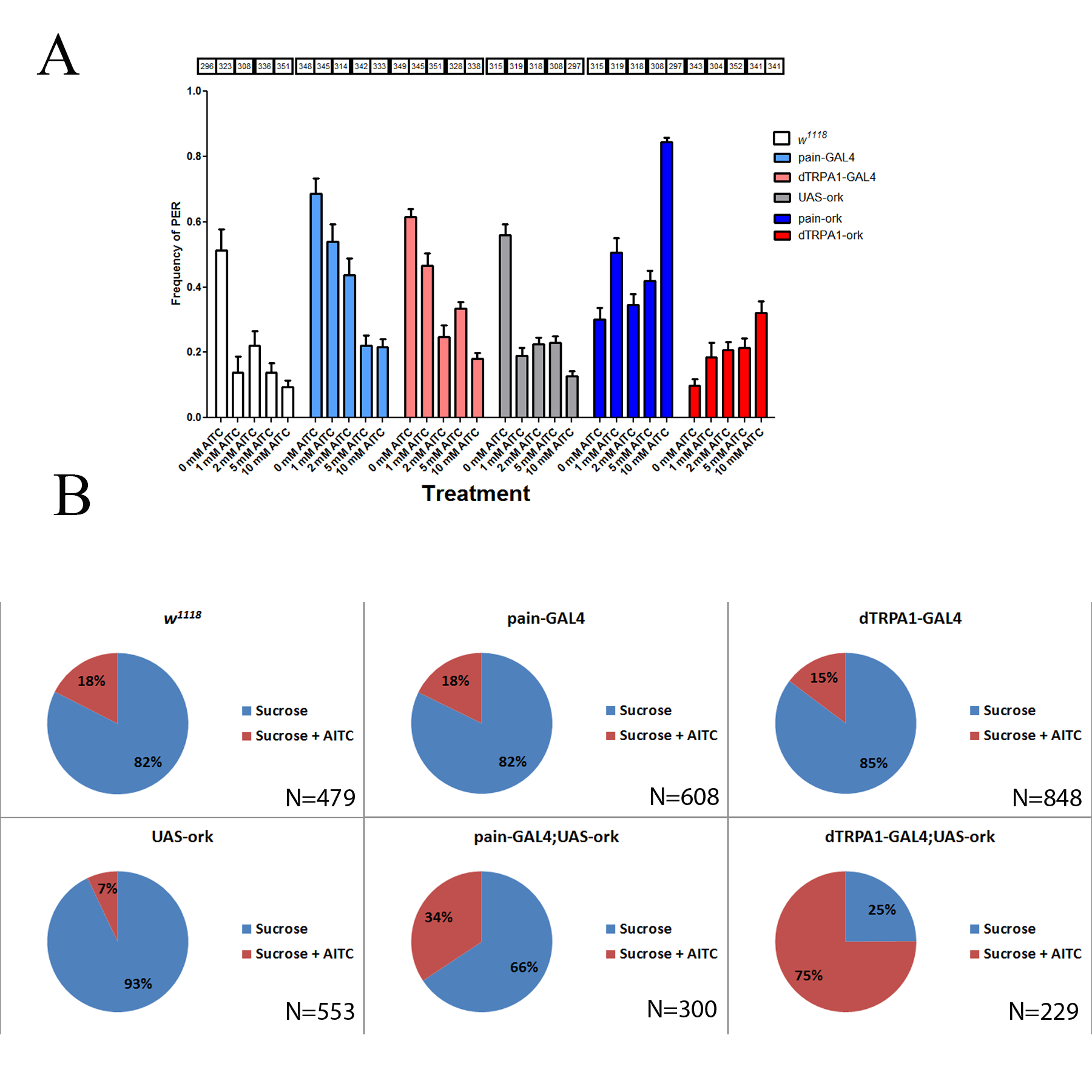

Supplement: FIGURE S3 — Electrical silencing of painless and dTRPA1 neurons impacts PER frequencies and oviposition preferences. (A) Electrical silencing of either the painless-GAL or the dTRPA1-GAL4 expressing neurons leads to no reduction in PER frequecies at 5% sucrose upon presentation of AITC as opposed to parental control genotypes (One way ANOVA, GraphPad). (B) Oviposition preference is lost in animals with silenced painless and dTRPA1 neurons, as equal levels of eggs were laid on AITC substrate as control substrate. [file Image_3.TIF]

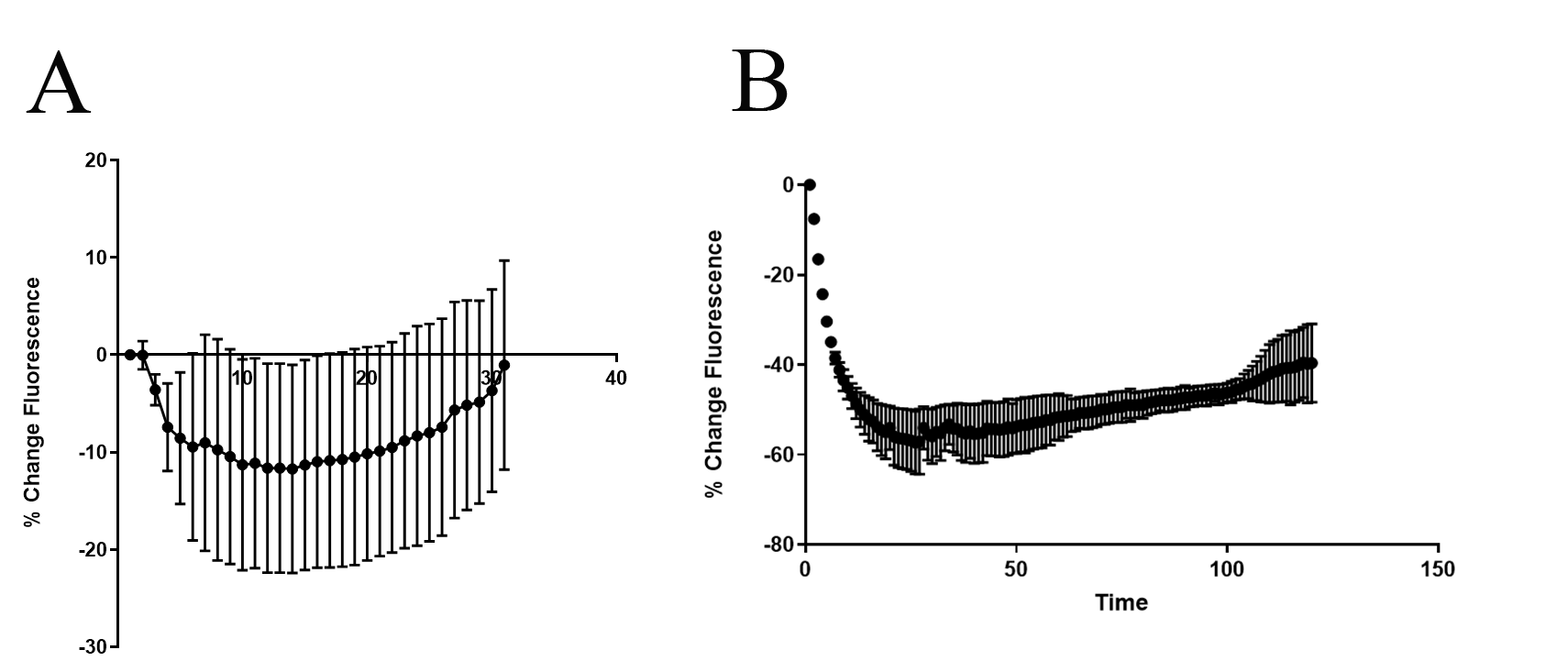

Supplement: FIGURE S4 — (A) Addition of vehicle to painless expressing neurons does not significantly alter GCaMP fluorescence. Mean responses of painless expressing neurons to vehicle addition, note the photobleaching and compare to responses shown in Figure 9. (B) Addition of AITC to neurons that do not express painless or dTRPA1 does not significantly alter GCaMP fluorescence. 2mM AITC application to neurons expressing GCaMP but not painless does not significantly change GCaMP fluorescence. Again note the photobleaching of GCaMP and compare to responses shown in Figure 9. [file Image_4.TIF]
